# Dead-time compensation in three-phase grid-tied inverters using LQG multivariable control

**DOI:** 10.1038/s41598-023-41944-2

**Published:** 2023-09-08

**Authors:** Ali Mazaheri, Farhad Barati, Farideh Ghavipanjeh

**Affiliations:** Department of Energy, Materials and Energy Research Centre, Karaj, Iran

**Keywords:** Photovoltaics, Wind energy, Batteries, Supercapacitors

## Abstract

Dead-time is the most important disturbance in a voltage-source inverter’s operation. It introduces low-order harmonics at the inverter’s output voltage. To compensate for the dead-time effects in three-phase grid-tied inverters, this paper proposes a Linear Quadratic Gaussian (LQG) multivariable control approach. The LQG multivariable control is known as a robust control approach while provides a high band-width for the closed-loop system. Therefore, it promises significant attenuations in the dead-time introduced harmonics. To achieve a high performance, we run the three-phase grid-tied inverter in the current-controlled mode. Based on the nominal multivariable model derived for the three-phase grid-tied inverter in a synchronous reference frame, the LQG controller is composed such that the closed-loop system exhibits robust stability while attenuates disturbances significantly. The dead-time introduced harmonics produce disturbances in the synchronous reference frame with the highest frequencies. This is the reason for considering the dead-time as the most important disturbance in an inverter’s operation. For an experimental set-up manufactured for the three-phase grid-tied inverter, we developed a detailed model in MATLAB/Simulink. It is employed for the performance verifications of designed LQG controller. Extensive results are presented for different important scenarios, based on which, the excellent performance of proposed approach is proven. In fact, by employing the proposed approach, the dead-time introduced harmonics are significantly attenuated such that a Total Harmonics Distortions (THD) of about 5% is achieved for the injected currents to grid which meets the IEEE 1547 standard.

## Introduction

Power electronics converters are essential components in renewable energy systems such as PV plants, wind power plants, and etc. They must provide high quality powers both at the AC and DC sides. The grid-tied inverter, as a power electronics converter, is the key component for the integration of renewable sources into the utility grid. Also, different renewable sources can be combined in the form of a DC microgrid which is connected to the utility grid through the grid-tied inverter. The DC microgrid includes energy storage elements too which are the balancing means between the generated powers of the renewable sources and the loads demand. Since the energy storage elements have limited capacities, at some points, the DC microgrid needs to exchange power with the utility grid which is done through the grid-tied inverter. In fact, the grid-tied inverter is able to provide the bi-directional power exchange between the DC microgrid and the utility grid. It acts either as an active rectifier when supplying the DC microgrid or as an inverter when injecting power to the utility grid. The grid-tied inverter’s operation mode as well as the amounts of power exchange required are determined by the DC microgrid’s supervisory control. Moreover, according to the IEEE 1547 standard, a Power Factor (PF) equals to or more than 0.8 is required for the grid-tied inverter at the Point of Common Couplings (PCC). A lagging PF supports the utility grid in terms of the reactive power^1,2^.

To have a desired performance, the grid-tied inverter needs to be equipped by a closed-loop control system. The closed-loop control system must provide robust stability as well as desired reference input tracking and disturbance rejection^3–5^. A three-phase grid-tied inverter is a Multiple-Input Multiple-Output (MI–MO) plant; meaning that each input affects all of the outputs. So, in order to achieve a suitable controller for the three-phase grid-tied inverter, multivariable analysis and design techniques must be employed. These techniques are obviously different from those applicable for Single-Input Single-Output (SI–SO) plants in which an input affects only one output. A suitable controller for the three-phase grid-tied inverter must compensate for the non-ideal conditions in the utility grid such as unbalanced conditions, distorted voltages, phase-jumping, frequency deviations, and etc^6–8^.

The H_∞_ and LQG are among the most advanced multivariable control techniques. They are able to deal well with unmodelled dynamics, uncertainties in the plant’s model parameters, and etc^9–11^. The LQG employs two optimal control problems including the Linear Quadratic Regulator (LQR) and the Kalman filter problems to synthesize a Model-Based Compensator (MBC). The resulting MBC provides robust stability as well as appropriate disturbance and noise rejections for the closed-loop control system^11–14^. The LQG may be followed by a Loop Transfer Recovery (LTR) to guarantee a suitable performance^15,16^.

An optimal LQG-based control is employed for the energy management system of all-electric vehicles^17^. The LQG is also employed for regulating active power flows in electric power systems^18^. In microgrids, the LQG control is utilized for enhancing dynamic response and optimal power flows^19,20^. An LQG robust control is utilized for power quality enhancement in a shunt-active power filter^21^ and for the current control of grid-tied inverters with the LCL filter^22,23^.

In^24,25^ active disturbance rejection technique is employed for grid-tied inverters. It is a model-free technique; meaning that it can be employed for a plant with unknown dynamics and disturbances. An equivalent input disturbance-based control is employed for three-phase grid-tied inverters considering the dead-time effects^26^.

**Figure 1:**
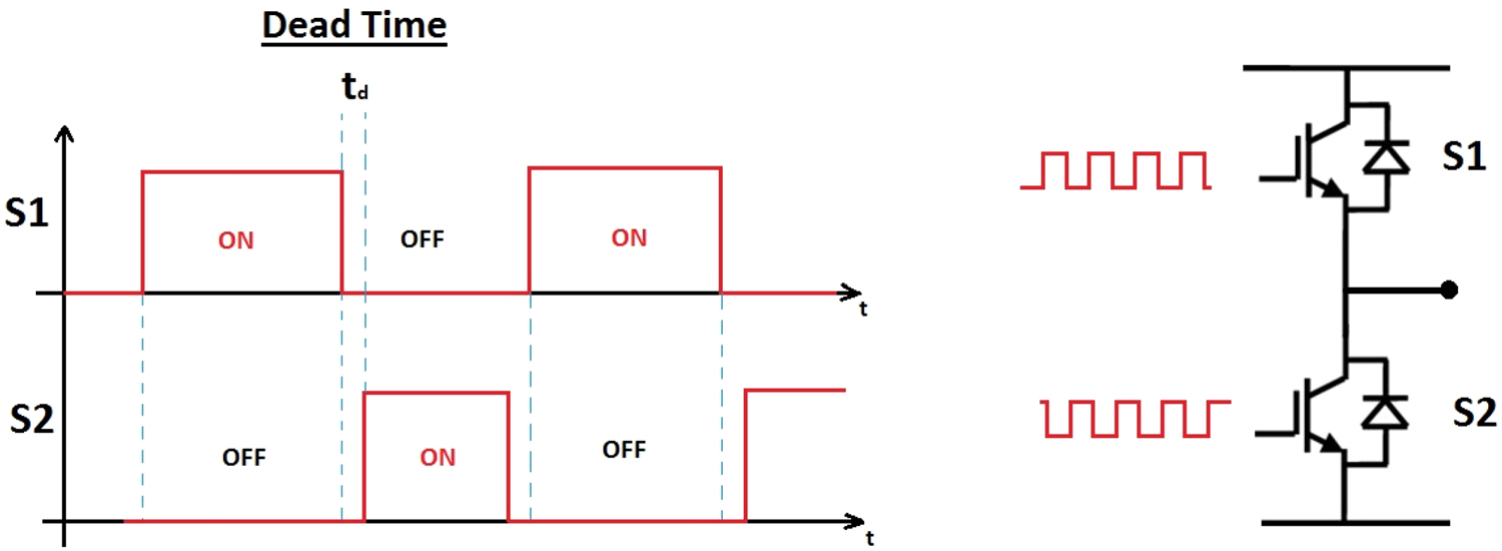
Definition of dead-time in an inverter’s leg.

In a two-level three-phase inverter, the switching signals are generated by a Sinusoidal Pulse-Width Modulator (SPWM) in which the controller’s outputs are compared with a high frequency sawtooth carrier. The frequency of sawtooth carrier equals to the switching frequency of power switches which are MOSFETs or IGBTs. An SPWM’s output is high when its corresponding controller’s output is higher than the carrier. In this case, the upper switch in the corresponding inverter’s leg is ON and the lower switch is OFF. When the SPWM’s output is low, the upper switch is OFF and the lower switch is ON. Since real power switches are not able to be turned-on or turned-off instantly, in order to prevent the short-circuit in an inverter’s leg, a dead-time is implemented in the SPWM. It is defined as in Fig. 1. By implementing the dead-time, the turning-on command for one switch in a leg is started when enough time, i.e., t_d_, elapsed from starting of the turning-off command of the other switch in the leg.

The inverter’s dead-time introduces low-order harmonics, i.e. the 5th, 7th, 11th, 13th, 17th, 19th,… harmonics, at the inverter’s output voltage^8^. At the result of the dead-time introduced harmonics, the inverter’s output current is heavily distorted. So, in an inverter’s operation, it is necessary to consider compensating for the dead-time effects.

In^8^ different strategies for mitigating the dead-time effects in power electronics converters are reviewed. They include pulse width adjustment, average voltage, current feedback, voltage feedback, and disturbance observer compensation methods. In the existing methods, in order to compensate for the dead-time effects, the robust stability of closed-loop control system is sacrificed. Therefore, there is the lack of a robust control technique for dead-time compensation in three-phase grid-tied inverters. The motivation of this paper is to compensate for the dead-time effects while the robust stability of closed-loop control system is maintained. For this purpose, in this paper, as the novel contribution, we propose an LQG-based multivariable control to compensate for the dead-time effects in three-phase grid-tied inverters. It employs the injected currents to grid feedback. No current direction detection circuit required, no feedforward terms employed, and no none-standard SPWM utilized in the proposed approach. We consider these as the advantages of the proposed approach over the existing methods. The main features of proposed approach are as follows.

- Besides the dead-time effects, it compensates for any low-order harmonics exists in the injected currents to grid originated from either the inverter, filter, or the grid.
- The closed-loop control system maintains the robust stability; meaning that it remains stable in spite of unmodelled dynamics and uncertainties.

## Three-phase grid-tied inverter

**Figure 2:**
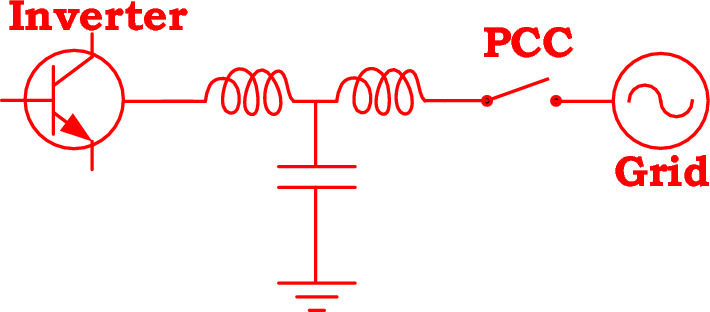
Grid-tied inverter with LCL filter.

**Figure 3:**
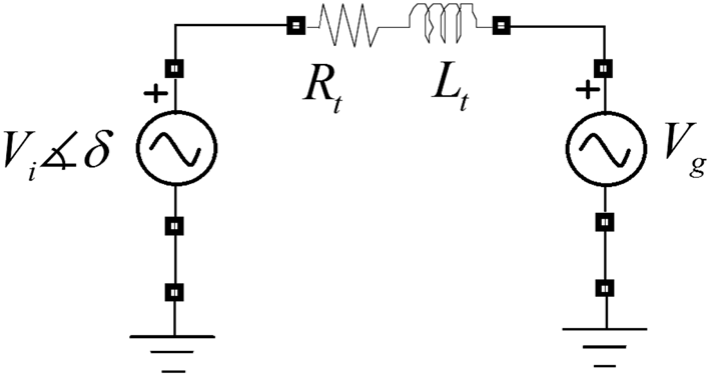
Circuit representation of grid-tied three-phase inverter’s vector model in a synchronous reference frame aligned with grid’s voltage vector.

A three-phase grid-tied inverter produces voltage harmonics at its outputs. In order to achieve sinusoidal current waveforms, the inverter’s outputs must pass through a filter. In Fig. 2, an LCL filter is employed for connecting the inverter to the grid at the PCC. The capacitor branch of the LCL filter provides a low impedance path for high-order harmonics. However, it shows high impedances at the fundamental frequency and low-order harmonics. An equivalent-circuit for the three-phase grid-tied inverter at the fundamental frequency is shown in Fig. 3. In this figure, $V_{g}$ is the grid’s voltage vector, $V_{i}$ is the inverter’s voltage vector, $R_{t}$ is the total resistance, and $L_{t}$ is the total inductance exist between the inverter and the grid. It is the circuit representation of the grid-tied three-phase inverter’s vector model in a synchronous reference frame. In this representation, the d-axis of the synchronous reference frame is aligned with the grid’s voltage vector. Based on Fig. 3, by decomposing the vector equations into the real and imaginary parts, the following equations can be derived^22^.

1 $$V_{id} = L_{t} \frac{{dI_{d} }}{dt} + R_{t} I_{d} - L_{t} \omega_{g} I_{q} + V_{g}$$

2 $$V_{iq} = L_{t} \frac{{dI_{q} }}{dt} + R_{t} I_{q} + L_{t} \omega_{g} I_{d}$$

where *ω*_*g*_ is the grid’s angular frequency. By defining $U_{d} = V_{id} - V_{g}$ and $U_{q} = V_{iq}$, the above equations can be re-written in the standard state-space representation as follows.

3 $$\left[ \begin{gathered} \dot{I}_{d} \hfill \\ \dot{I}_{q} \hfill \\ \end{gathered} \right] = \underbrace {{\left[ {\begin{array}{*{20}c} { - \frac{{R_{t} }}{{L_{t} }}} & {\omega_{g} } \\ { - \omega_{g} } & { - \frac{{R_{t} }}{{L_{t} }}} \\ \end{array} } \right]}}_{{A_{p} }}\left[ \begin{gathered} I_{d} \hfill \\ I_{q} \hfill \\ \end{gathered} \right] + \underbrace {{\left[ {\begin{array}{*{20}c} {\frac{1}{{L_{t} }}} & 0 \\ 0 & {\frac{1}{{L_{t} }}} \\ \end{array} } \right]}}_{{B_{p} }}\left[ \begin{gathered} U_{d} \hfill \\ U_{q} \hfill \\ \end{gathered} \right]$$

4 $$\left[ \begin{gathered} I_{d} \hfill \\ I_{q} \hfill \\ \end{gathered} \right] = \underbrace {{\left[ {\begin{array}{*{20}c} 1 & 0 \\ 0 & 1 \\ \end{array} } \right]}}_{{C_{p} }}\left[ \begin{gathered} I_{d} \hfill \\ I_{q} \hfill \\ \end{gathered} \right] + \underbrace {{\left[ {\begin{array}{*{20}c} 0 & 0 \\ 0 & 0 \\ \end{array} } \right]}}_{{D_{p} }}\left[ \begin{gathered} U_{d} \hfill \\ U_{q} \hfill \\ \end{gathered} \right]$$

According to the above representation, it is clear that the grid-tied three-phase inverter is a MI–MO system with two inputs and two outputs. In fact, the vector $$\left[ {\begin{array}{*{20}c} {U_{d} } & {U_{q} } \\ \end{array} } \right]^{T}$$ is the input vector and the vector $$\left[ {\begin{array}{*{20}c} {I_{d} } & {I_{q} } \\ \end{array} } \right]^{T}$$ is the output vector. One can derive the transfer matrix for the three-phase grid-tied inverter as follows.

5 $$G_{p} (s) = \frac{1}{{L_{t} }}\frac{{\left[ {\begin{array}{*{20}c} {s + \frac{{R_{t} }}{{L_{t} }}} & {\omega_{g} } \\ { - \omega_{g} } & {s + \frac{{R_{t} }}{{L_{t} }}} \\ \end{array} } \right]}}{{s^{2} + 2\frac{{R_{t} }}{{L_{t} }}s + ((\frac{{R_{t} }}{{L_{t} }})^{2} + \omega_{g}^{2} )}}$$

The above relation shows that the grid-tied three-phase inverter in the synchronous reference frame is a second-order system with two poles in the left half-plane. Also, it can be shown that the $$\begin{array}{*{20}c} {(A_{p} } & {B_{p} )} \\ \end{array}$$ pair is controllable and the $$\begin{array}{*{20}c} {(A_{p} } & {C_{p} )} \\ \end{array}$$ pair is observable and the plant is a minimum-phase one. Therefore, the three-phase grid-tied inverter has the required conditions to be controlled using the LQG multivariable controller^27^.

## Multivariable control systems analysis and LQG control design

### Multivariable control systems analysis

**Figure 4:**
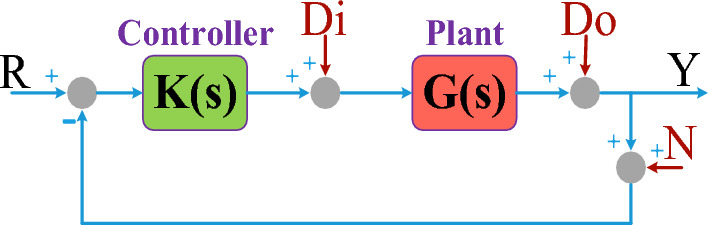
A generic closed-loop control system.

Figure 4 illustrates a general structure for a closed-loop control system where, R is the reference input, Y is the output, N is the measurement noise, D_i_ is the plant’s input disturbance, and D_o_ is the plant’s output disturbance. In MI–MO systems, R and Y are two vectors of equal dimensions. In addition to the closed-loop system stability, its performance must be desired in terms of the reference input tracking and noise and disturbance rejections. For a MI–MO system with *n* inputs and *n* outputs, the performance criteria can be stated as follows^27^.

1. *Reference input tracking* In $Y(s) = \left[ {(I + G(s)K(s))^{ - 1} G(s)K(s)} \right]R(s)$, for $\omega \in \omega_{R}$, if $G(s)K(s)$ >  > $I$ is satisfied, then $Y(s) \approx R(s)$ is achieved. $\omega \in \omega_{R}$ means all the frequencies at which the elements of *R(s)* have significant energies.
2. *Input disturbance rejection* In $Y(s) = \left[ {(I + G(s)K(s))^{ - 1} G(s)} \right]D_{i} (s)$, for $\omega \in \omega_{{D_{i} }}$, if $G(s)K(s)$ >  > *I* and $K(s)$ >  > *I* are satisfied simultaneously, then $Y(s) \approx 0$ is achieved. $\omega \in \omega_{{D_{i} }}$ means all the frequencies at which the elements of *D*_*i*_*(s)* have significant energies.
3. *Output disturbance rejection* In $Y(s) = \left[ {(I + G(s)K(s))^{ - 1} } \right]D_{o} (s)$, for $\omega \in \omega_{{D_{o} }}$, if $G(s)K(s)$ >  > *I* is satisfied, then $Y(s) \approx 0$ is achieved. $\omega \in \omega_{{D_{o} }}$ means all the frequencies at which the elements of *D*_*o*_*(s)* have significant energies.
4. *Noise rejection* In $Y(s) = \left[ { - (I + G(s)K(s))^{ - 1} G(s)K(s)} \right]N(s)$ , for $\omega \in \omega_{N}$ , if $G(s)K(s)$ <  < *I* is satisfied, then $Y(s) \approx 0$ is achieved. $\omega \in \omega_{N}$ means all the frequencies at which the elements of *N(s)* have significant energies.

The reference inputs and input and output disturbances usually have most of their energies at low frequencies while most of the measurement noise’s energy is located at high frequencies. Based on the above, we can conclude that sufficient conditions for satisfying all of the closed-loop control system performance criteria are as follows^27^.

- *At low frequencies:* $K(s)$ >  > *I* and $G(s)K(s)$ >  > *I* are satisfied simultaneously.
- *At high frequencies:* $G(s)K(s)$ <  < *I* is satisfied.

### Singular value decomposition

As can be seen in the above, we need to compare a given matrix with the identity matrix, i.e. *I*. To do so, the Singular Value Decomposition (SVD) technique is employed^27^. In the SVD, a given matrix A is decomposed into three matrices as follows. For $A_{m \times n}$, we have:

6 $$A = U\sum \,\,V^{T} \,$$

where U is an $m \times m$ unitary matrix, i.e. $UU^{T} = I$, and V is an $n \times n$ unitary matrix too, i.e. $VV^{T} = I$. The $\sum$ is an $m \times n$ rectangular diagonal matrix with $\sigma_{i}$ as the $i^{th}$ singular value of matrix A on the diagonal. We define $\overline{\sigma }$ as the largest singular value of A and $\underline {\sigma }$ as the smallest singular value of matrix A.

Based on the SVD technique, in order to satisfy the condition $G(s)K(s)$ <  < *I*, it is sufficient that the condition $\overline{\sigma }\left[ {G(s)K(s)} \right]$ <  < 1 be satisfied. Also, to satisfy the condition $G(s)K(s)$ >  > *I*, it is sufficient that the condition $\underline {\sigma } \left[ {G(s)K(s)} \right]$ >  > 1 be satisfied.

### LQG control design

The LQG consists of two optimal control problems including the LQR and the Kalman filter problems which the former is employed for the state feedback design and the latter is employed for the state estimation^27^. We consider the state-space representation of the plant as follows.

7 $$\begin{aligned} \dot{X} &= A_{p} X + B_{p} U + \Gamma w \hfill \\ Y &= C_{p} X + D_{p} U + v \hfill \\ \end{aligned}$$

where $A_{p}$, $B_{p}$, $C_{p}$, and $D_{p}$ are the plant’s state-space representation matrices. Also, *w* and *v* are assumed to be white noises which the former is considered as the process noise and the latter is considered to be the measurement noise. Their associated covariance matrices are shown as follows.

8 $$E\left\{ {ww^{T} } \right\} = N > 0 \quad E\left\{ {vv^{T} } \right\} = G > 0 \quad E\left\{ {wv^{T} } \right\} = 0$$

For the LQR, noises are not included in which we want to obtain an optimal gain for the full state variables feedback. The gain must minimize the following quadratic cost function.

9 $$J = \int\limits_{0}^{\infty } {\left[ {X^{T} QX + U^{T} RU} \right]} \,dt$$

where R and Q are arbitrary positive-definite matrices. In LQR, we choose $U = - K_{LQR} \,X$. It can be shown that $K_{LQR} = R^{ - 1} B_{p}^{T} P_{LQR}$ where, *P*_*LQR*_ is obtained from the Algebraic Riccati Equation (ARE) as follows.

10 $$A_{p}^{T} P_{LQR} + P_{LQR} A_{p} - P_{LQR} B_{p} R^{ - 1} B_{p}^{T} P_{LQR} + Q = 0$$

In the Kalman filter, noises are included in the plant’s model. The Kalman filter gain is chosen such that the following cost function in minimized.

11 $$J = E\left\{ {\tilde{X}^{T} \tilde{X}} \right\}$$

where $\tilde{X} = \hat{X} - X$ is the state estimation error and $\hat{X}$ is the estimation of state vector $X$. One can obtain the Kalman filter gain as $K_{Kalman} = P_{Kalman} C_{p}^{T} G^{ - 1}$ where, $P_{Kalman}$ is obtained from the following Filter Algebraic Riccati Equation (FARE).

12 $$P_{kalman} A_{p}^{T} + A_{p} P_{kalman} - P_{kalman} C_{p}^{T} G^{ - 1} C_{p} P_{Kalman} + \Gamma N\Gamma^{T} = 0$$

Based on the above, the multivariable LQG controller is composed as follows.

13 $$K(s) = K_{LQR} (sI - A_{p} + B_{p} K_{LQR} + K_{Kalman} C_{p} )^{ - 1} K_{Kalman}$$

By employing the LQG controller, it can be shown that, the closed-loop system is stable for the nominal plant. It is also confirmed that the closed-loop control system with the LQG controller exhibits the robust stability^27^. However, its performance depends on the controller’s free parameters, i.e. $K_{LQR}$ and $K_{Kalman}$. We tune the free parameters such that the closed–loop control system performance is desired in terms of the reference inputs following and disturbances and noise rejections. In fact, to tune $K_{LQR}$, we have to choose Q and R properly which are symmetric positive semi-definite and positive definite matrices, respectively. Also, to tune $K_{Kalman}$, the matrices $N$ and $G$ must be properly chosen.

The maximum and minimum singular values of the loop transfer matrix, i.e. *G(s) K(s)*, as well as the controller, i.e. *K(s)*, versus the frequency are employed for the designed controller’s evaluations. These diagrams are called the SVD diagrams. In fact, we select the $K_{LQR}$ and $K_{Kalman}$ simultaneously. So, the impact of $K_{LQR}$ and $K_{Kalman}$, as a pair, is on the SVD diagrams. The SVD diagrams show the capability of designed controller in supressing the harmonics and in providing the robust stability. A higher SVD diagrams for the loop transfer matrix and for the controller in the low frequency range indicate a higher performance controller especially in terms of harmonics suppressions. Also, a lower SVD diagram for the loop transfer matrix in the high frequency range indicates a more robust controller.

## Proposed dead-time compensation method

**Figure 5:**
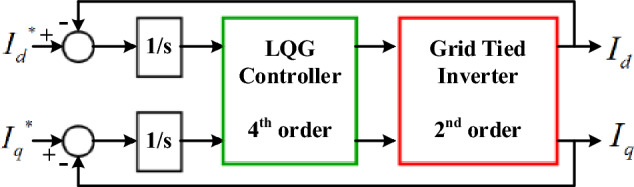
Proposed dead-time compensation method.

The proposed method of this paper for dead-time compensation in three-phase grid-tied inverters is shown in Fig. 5. As shown, it employs the LQG multivariable control for regulating the injected currents to grid in the synchronous reference frame. To have the zero steady-state error in response to the step reference inputs, an integrator must be added at each of the plant’s inputs. Therefore, the yielding system under control is of the 4th order which is the order of the LQG controller too. In the other words, the order of the LQG controller equals to the order of the system under control.

### Proposed method analysis

Besides the dead-time, the proposed method must deal well with harmonics in the grid’s voltages. In fact, both of the dead-time introduced harmonics and grid’s voltages harmonics are low-order harmonics. This is in contrast with the SPWM’s harmonics which are high-order ones. The SPWM’s harmonics are well suppressed by employing the LCL filter. So, in terms of the injected currents to grid, their effects can be ignored. The LCL filter behaves such as an L filter for low-order harmonics, since the capacitor branch can be considered as open-circuited at low frequencies. Since small inductors are utilized in the LCL filter, the resulting L filter at low frequencies is not able to significantly suppress the low-order harmonics. Therefore, this is the closed-loop control system’s responsibility to attenuate low-order harmonics as much as possible such that the THD of injected currents to grid meets the IEEE 1547 standard requirements.

For a three-phase grid-tied inverter in the synchronous reference frame, low-order harmonics in the injected currents to grid result in disturbances in $I_{d}$ and $I_{q}$. As stated before, the inverter’s dead-time introduces the 5th, 7th, 11th, 13th, 17th, 19th,… harmonics at the inverter’s output voltage. Therefore, it introduces disturbances at $6\omega_{g}$ frequency and its multiples, i.e. $12\omega_{g}$, $18\omega_{g}$, …, in $I_{d}$ and $I_{q}$ where, *ω*_*g*_ is the grid’s angular frequency. In fact, the derived vector model for the three-phase grid-tied inverter described in "Three-phase grid-tied inverter" section is in the synchronous reference frame aligned with the grid’s voltage vector. In the case of an un-balanced grid, the reference frame is aligned with the positive-sequence voltage vector. The positive-sequence voltage vector is rotating at *ω*_*g*_ angular speed with respect to the stationary reference frame. Considering the 5th and 7th harmonics, the 5th harmonics produces a negative-sequence voltage vector rotating at − 5*ω*_*g*_ angular speed with respect to the stationary reference frame. So, in the reference frame rotating at *ω*_*g*_ angular speed, it is producing a 6*ω*_*g*_ disturbance. On the other hand, the 7th harmonics produces a positive-sequence voltage vector rotating at 7*ω*_*g*_ angular speed with respect to the stationary reference frame. So, in the reference frame rotating at *ω*_*g*_ angular speed, the 7th harmonics produces a 6*ω*_*g*_ disturbance too. Therefore, the 6*ω*_*g*_ disturbance in the synchronous reference frame is at the result of both the 5th and 7th dead-time introduced harmonics. Note that, the amplitudes of disturbances are proportional to the amounts of dead-time considered. Also, they are inversely proportional to the frequency of the disturbance.

An un-balanced grid introduces disturbances at $2\omega_{g}$ frequency in $I_{d}$ and $I_{q}$ which are the effects of the negative-sequence component exists in an un-balanced grid. It is, therefore, clear that the inverter’s dead-time introduces disturbances with the highest frequencies in $I_{d}$ and $I_{q}$. So, they are the most important disturbances in an inverter’s operation.

### Proposed method design

We define *G(s)* = *G*_*p*_*(s)G*_*a*_*(s)* as the new plant where, *G*_*a*_*(s)* is the transfer matrix of the two augmented integrators at the plant’s inputs. The LQG MBC is composed according to the equation in (13) and the procedure described for choosing its free parameters, i.e. $K_{LQR}$ and $K_{Kalman}$. We design the LQG controller such that *G(s)K(s)* >  > *I* and $K(s)$ >  > *I* are satisfied simultaneously for the low frequency range up to the $6\omega_{g}$ frequency. Note that, the $6\omega_{g}$ frequency is the first dead-time introduced disturbance and it has the highest amplitude. This guarantees appropriate reference inputs tracking and disturbance rejection. It, of course, covers the $2\omega_{g}$ frequency disturbance too which is the un-balanced grid introduced disturbance.

An experimental set-up, as shown in the Online Appendix, is manufactured for the purposes of implementing and verifying the proposed method. In Table 1, its parameters are listed. A three-phase BSM50GP120 IGBT module is employed in the experimental set-up. We considered the switching frequency of $f_{s}$ = 20 kHz for the IGBT power switches. An LCL filter with the parameters listed in Table 1 is designed to suppress the SPWM’s harmonics. The inverter side inductor, *L*_*i*_, and the grid side inductor, *L*_*g*_, are wound on two separate toroidal cores for which the windings’ resistances are measured. The *L*_*t*_ = *L*_*i*_ + *L*_*g*_ and *R*_*t*_ is the summation of the inverter side and grid side inductors’ windings resistances.

**Table 1:** Parameters of the manufactured experimental set-up for three-phase grid-tied inverter.

Parameter	Symbol	Value
IGBT module	BSM50GP120	–
IGBT’s driver IC	IR2213	–
Switching frequency	$f_{s}$	20 kHz
Inverter side inductance	*L*_*i*_	0.3 mH
Grid side inductance	*L*_*g*_	0.2 mH
Summation of resistances of inverter side and grid side inductances	*R*_*t*_	0.285 $\Omega$
Filter capacitor	*C*_*f*_	4.7 µF
Filter damping resistor	*R*_*f*_	1 Ω
Inverter’s DC link voltage	*V*_*DC*_	750 V
Nominal voltage of three-phase grid	*V*_*g*_ (L − L)	400 *Vrms*
Inverter’s apparent power	*S*	10 KVA
Grid’s angular frequency	*ω*_*g*_	100π rad/s
Inverter’s dead-time	t_d_	1.5 µs

**Figure 6:**
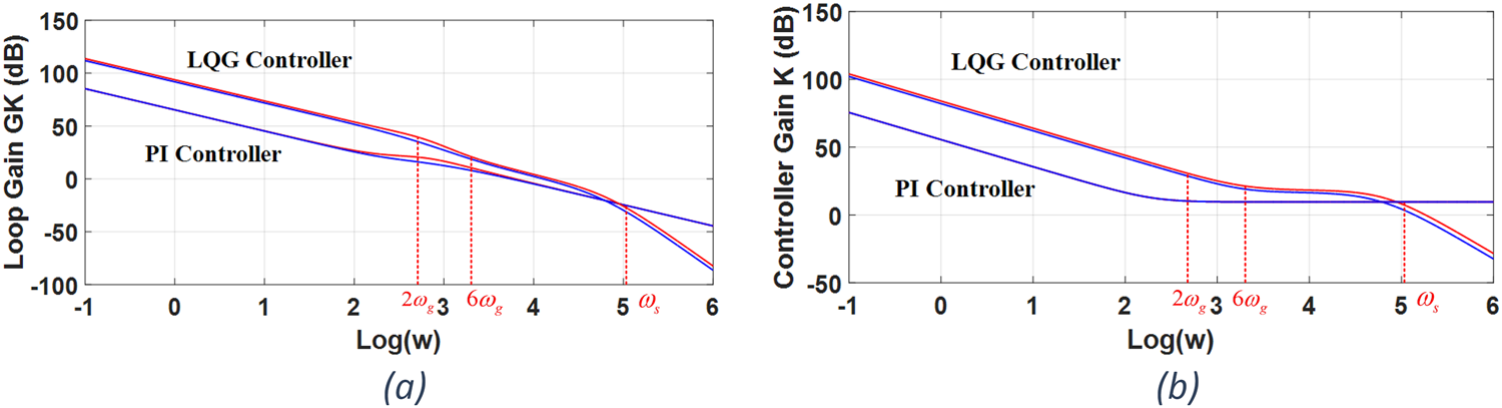
SVD diagrams of (**a**) loop transfer matrix and (**b**) controller for LQG and PI controllers.

According to the turn-on and turn-off times of the IGBT power switches as well as the IGBT’s driver IC, a dead-time equals to *t*_*d*_ = 1.5 µs is calculated which guarantees that no short-circuit occurs in an inverter’s leg. Note that, in order to minimize its adverse effects, the dead-time calculated is the minimum possible one. It is implemented in a three-phase SPWM to command the three legs of inverter. For the given new plant, the LQG controller is designed and the results are shown in Fig. 6. In Fig. 6, the SVD diagrams are shown both for the loop transfer matrix, i.e. *G(s)K(s)*, and the LQG controller, i.e. *K(s)*. For the purpose of comparison, the results for a multivariable PI controller as presented in^28^ are shown too.

As stated before, to have the desired performance, it is sufficient to have $\underline {\sigma }$ >  > 1 in the low frequency range up to the $6\omega_{g}$ frequency both for the loop transfer matrix and the controller. Also, for the frequency range above the switching angular frequency, i.e. ω_s_, it is sufficient to have $\overline{\sigma }$ <  < 1 for the loop transfer matrix. Note that, we considered the frequency range above the switching angular frequency as the high frequency range in this study. This is due to the fact that the capacitor branch’s effects which are ignored in the three-phase grid-tied inverter’s nominal model development become important at the switching angular frequency and above it.

According to Fig. 6, in the low frequency range up to the $6\omega_{g}$ frequency, the designed LQG controller provides a minimum singular value much higher than the unity both for the loop transfer matrix and the controller. This guarantees a very well reference inputs tracking as well as disturbance rejections for the closed-loop control system. Also, in the high frequency range, the designed LQG controller provides a maximum singular value much smaller than the unity for the loop transfer matrix. This guarantees that the LCL filter’s capacitor branch and other unmodelled dynamics, which are usually significant at high frequencies, will have no adverse effects on the closed-loop control system stability.

The SVD diagrams for the multivariable PI controller are lower in the low frequency range and higher in the high frequency range than those of the LQG controller as clear in Fig. 6. Therefore, a much higher disturbances suppressions and a better robust stability are expected for the designed LQG controller.

## Results

**Figure 7:**
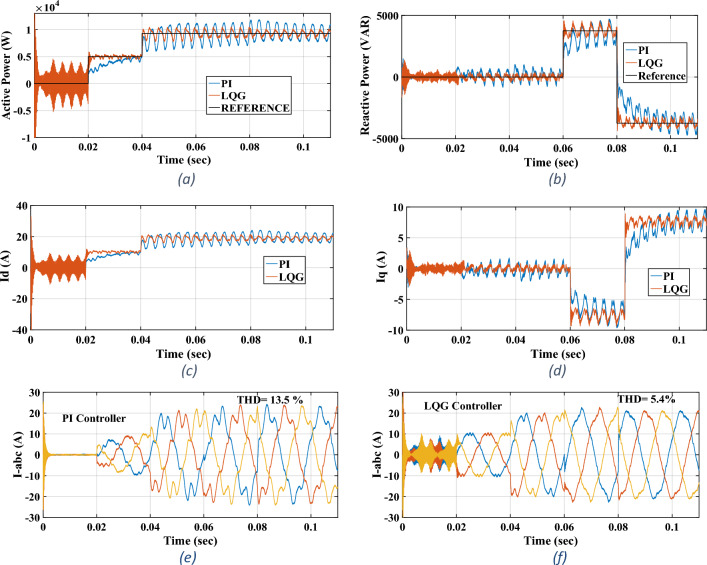
Reference inputs tracking test (**a**) active power (**b**) reactive power (**c**) Id (**d**) Iq (**e**) three-phase currents for PI controller (**f**) three-phase currents for LQG controller.

**Figure 8:**
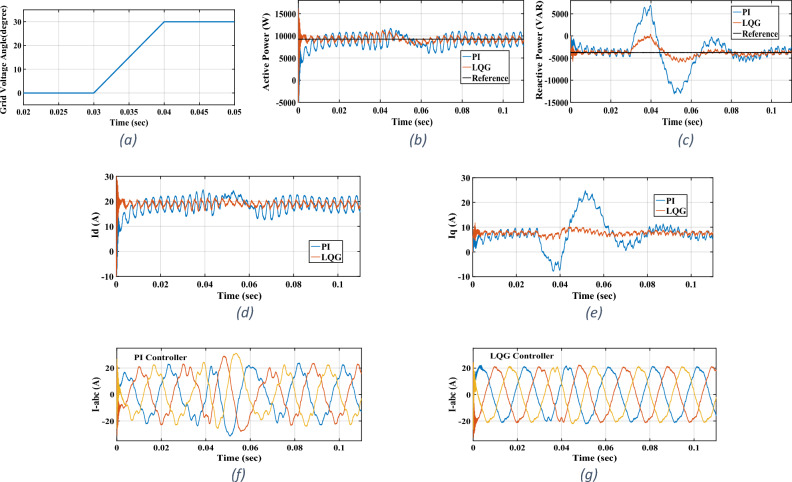
Phase-jumping test: (**a**) phase A voltage angle, (**b**) active power, (**c**) reactive power, (**d**) I_d_, (**e**) I_q_, (**f**) three-phase currents for PI controller, (**g**) three-phase currents for LQG controller.

**Figure 9:**
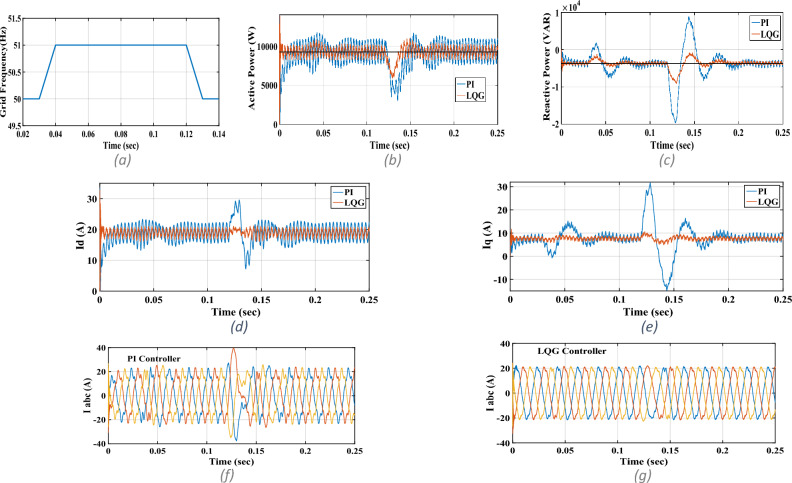
Grid frequency deviations test: (**a**) grid frequency, (**b**) active power, (**c**) reactive power, (**d**) I_d_, (**e**) I_q_, (**f**) three-phase currents for PI controller, (**g**) three-phase currents for LQG controller.

**Figure 10:**
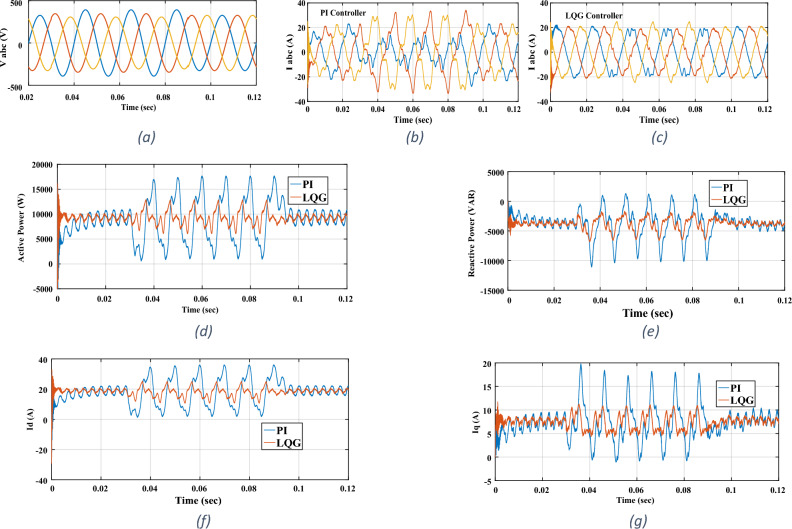
Un-balanced grid test: (**a**) grid’s three-phase voltages (**b**) three-phase currents for PI controller (**c**) three-phase currents for LQG controller (**d**) active power (**e**) reactive power (**f**) I_d_ **g**) I_q_.

For the experimental set-up manufactured for the three-phase two-level grid-tied inverter, we developed a detailed model in MATLAB/Simulink environment. In fact, the developed model consists of the two-level three-phase inverter with IGBT power switches, the LCL filter, the utility grid, a Phase-Locked Loop (PLL), the SPWM, and the closed-loop control system. It is shown in the Online Appendix. We performed the following tests on the detailed model and the results are shown in Figs. 7, 8, 9 and 10. In each test, for the purpose of comparison, the results for the multivariable PI controller as presented in^28^ are shown too.

### Reference inputs tracking

The performance of closed-loop control system is evaluated in terms of following the reference inputs in this test. In fact, the reference inputs for the current closed-loop control system are calculated from the reference values for active and reactive powers injected to the grid according to the following equations.

14 $${\text{P}}_{{{\text{ref}} }} = {1}.{5}\left( {{\text{V}}_{{{\text{gd}}}} {\text{I}}^{*}_{{\text{d}}} + {\text{ V}}_{{{\text{gq}}}} {\text{I}}^{*}_{{\text{q}}} } \right)$$

15 $${\text{Q}}_{{{\text{ref}} }} = {1}.{5}\left( { - {\text{V}}_{{{\text{gd}}}} {\text{I}}^{*}_{{\text{q}}} + {\text{ V}}_{{{\text{gq}}}} {\text{I}}^{*}_{{\text{d}}} } \right)$$

where $V_{gd} = V_{g}$ and $V_{gq} = 0$ for the synchronous reference frame aligned with the grid’s voltage vector. Also, *V*_*g*_ is the grid’s phase voltage amplitude. Note that, P_ref_ and Q_ref_ are received from the supervisory control in a grid-tied DC microgrid.

In Fig. 7, as seen, the reference value of active power is changed at *t* = 0.02 s and t = 0.04 s. Also, the reference value of reactive power is changed at *t* = 0.06 s and *t* = 0.08 s. The reference values for *I*_*d*_ and *I*_*q*_ are changed accordingly. As an important performance criterion for the closed-loop control system, these reference values for *I*_*d*_ and *I*_*q*_ must be appropriately followed by the actual currents. As clear, at steady-states, the mean values of *I*_*d*_ and *I*_*q*_ equal to their reference values. So, in terms of the mean values, the steady-state errors are zero. However, there exists oscillating components in the instantaneous *I*_*d*_ and *I*_*q*_ with the frequency of 6*ω*_*g*_. We know that the 6*ω*_*g*_ frequency is introduced by the inverter’s dead-time whose effects are supposed to be suppressed by the proposed approach. As clear, an adequate suppression is performed resulting in the THD = 5.4% for the injected currents to grid by employing the proposed approach. The multivariable PI controller, however, results in the THD = 13.5% for the injected currents to grid. Note that, the THD is calculated for the nominal apparent power which, in this test, corresponds to P_ref_ = 9.27 kW and Q_ref_ = − 3.75 kVAR. This condition means a leading PF equals to PF = 0.927 which is more than 0.8. Note that, the leading PF means that the inverter absorbs some reactive power from the utility grid.

### Phase-jumping

The phase-jumping is common in the utility grid in which the phase angles of the three-phase grid change suddenly in a symmetrical manner. The PLL must appropriately follow the grid’s voltage vector angle; otherwise, the inverter loses its synchronism with the utility grid. The PLL dynamics, however, has not been considered in the nominal model derived for the three-phase grid-tied inverter. So, its dynamics is considered as an unmodelled one in this study. In this test with P_ref_ = 9.27 kW and Q_ref_ = − 3.75 kVAR, the effects of PLL dynamics in following the grid’s voltage vector angle are studied. For this purpose, at *t* = 0.03 s, we introduced a balanced phase-jumping in the grid’s three-phase voltages. As seen in Fig. 8, transients occur in the actual active and reactive powers and also in *I*_*d*_ and *I*_*q*_. The severest transients occur in the actual reactive power and in *I*_*q*_. As seen, transients have small amplitudes; meaning that they are suppressed by the LQG controller well. This fact is made clear when the results of LQG controller are compared with those of the multivariable PI controller.

### Deviations in the grid frequency

In the utility grid, deviations often occur in the frequency from the nominal frequency. So, the same as the phase-jumping, it is important that the PLL follows the grid frequency appropriately.

n this test with P_ref_ = 9.27 kW and Q_ref_ = − 3.75 kVAR, the effects of PLL dynamics in following the grid’s frequency are studied. For this purpose, the grid frequency is increased from 50 to 51 Hz and then decreased from 51 to 50 Hz as shown in Fig. 9. As can be seen, transients occur which the severest are, again, in the actual reactive power and in *I*_*q*_. It is clear that the transients are well suppressed by the LQG controller while the grid frequency deviations introduce large transients in the case of the multivariable PI controller.

### Unbalanced grid

An un-balanced condition is common in the utility grid. As stated before, it introduces disturbances at $2\omega_{g}$ frequency in *I*_*d*_ and *I*_*q*_. In this test, for P_ref_ = 9.27 kW and Q_ref_ = − 3.75 kVAR, an un-balanced condition is introduced from *t* = 0.03 s to *t* = 0.09 s for three grid cycles. The condition corresponds to a 5% of negative-sequence component in the grid’s voltages, i.e. + 20% change in the phase A amplitude, + 6% change in the phase B amplitude, and − 6% change in the phase C amplitude. Note that, the percentage of negative-sequence component is calculated with respect to the positive-sequence component. The results are shown in Fig. 10. As seen, by employing the LQG controller, the three-phase currents are close to balanced ones. This means that the $2\omega_{g}$ frequency disturbances are well suppressed by the LQG controller such that the negative-sequence component in the injected currents to grid is negligible. This fact is made clearer when the results of LQG controller are compared with those of the multivariable PI controller. Note that, in *t* = 0.03 s to *t* = 0.09 s interval, there exists two disturbances in *I*_*d*_ and *I*_*q*_ with $2\omega_{g}$ frequency and 6*ω*_*g*_ frequency which the former is introduced by the un-balanced condition and the latter is introduced by the dead-time.

## Discussions

In this section, we present a discussion about the proposed approach according to the results obtained. Note that, in this study, the frequency range up to the 6*ω*_*g*_ frequency is defined as the low frequency range and the frequency range above the *ω*_*s*_ frequency is defined as the high frequency range.

### Classifications of different phenomena in three-phase grid-tied inverter

The three-phase grid-tied inverter includes three main subsystems. They are the inverter, the filter, and the grid. Different phenomena exist in these three subsystems as classified in Table 2. In Table 2, for each phenomenon, it is made clear that how the phenomenon is considered and how it is resolved in the proposed approach.

**Table 2:** Classifications of different phenomena in three-phase grid-tied inverter.

Subsystem	Phenomenon	How considered in the proposed approach	How resolved in the proposed approach
Inverter	Dead-time	As disturbances	For the low frequency range *G(s)K(s)* > > *I* and *K(s)* > > *I* are satisfied simultaneously
	SPWM’s harmonics	As noises	For the high frequency range *G(s)K(s)* < < *I* is satisfied
	PLL	As an unmodelled dynamics	
Filter	Capacitor branch	As an unmodelled dynamics	
Grid	Harmonics	As disturbances	For the low frequency range *G(s)K(s)* > > *I* and *K(s)* > > *I* are satisfied simultaneously
	Unbalanced conditions	As disturbances	
	Variation in inductance	As an uncertainty	For the high frequency range *G(s)K(s)* < < *I* is satisfied

For the inverter subsystem which includes both of the power circuit and the control system, the dead-time, SPWM’s harmonics, and PLL are considered as the main phenomena. For the LCL filter, we consider the capacitor branch as the main phenomenon. Also, for the utility grid, we consider harmonics, un-balanced conditions, phase-jumping and frequency deviations, and variations in the grid’s inductance as the main phenomena.

As shown in Table 2, in the proposed approach, we consider the dead-time as the disturbances with the highest frequencies. Among all of the dead-time introduced disturbances, we considered the 6*ω*_*g*_ frequency disturbance in the LQG controller’s design procedure which is the first one and has the highest amplitude. As the highest frequency disturbance considered in the LQG controller’s design, considerable suppression is provided at the 6*ω*_*g*_ frequency in the proposed approach. The SPWM’s harmonics are well suppressed by the LCL filter; meaning that their effects are negligible in the injected currents to grid as seen in the results. In the proposed approach, however, we consider the effects of SPWM’s harmonics in the injected currents to grid as noises which fall in the high frequency range, i.e. above *ω*_*s*_. We know that the proposed approach provides significant attenuations in the high frequency range. The PLL and the LCL filter’s capacitor branch are the two main unmodelled dynamics in this study. As a matter of fact, unmodelled dynamics are significant at high frequencies. Therefore, in order to prevent the adverse effects of unmodelled dynamics on the closed-loop control system stability, it is sufficient that the loop transfer matrix be much smaller than the identity matrix in the high frequency range^27^. As seen in "Proposed dead-time compensation method" section, the proposed approach satisfies the mentioned sufficient condition. So, the unmodelled dynamics in this study are well suppressed such that the closed-loop control system does not exhibit instability as clear in the results presented in the previous section.

Harmonics in the three-phase grid’s voltages are low-order ones. So, they can not be significantly attenuated by the LCL filter. Therefore, they can produce significant low-order harmonics in the injected currents to grid. Low-order harmonics in the injected currents to grid which are originated from either the inverter, grid, or filter produce disturbances in *I*_*d*_ and *I*_*q*_. According to the proposed approach, disturbances whose frequencies fall in the low frequency range, as defined in this study, are significantly suppressed. It is also clear that the un-balanced condition which produces disturbances at the 2*ω*_*g*_ frequency is suppressed well.

The grid’s frequency deviations and phase-jumping cause transients in the grid’s voltage vector angle calculated by the PLL. These transients, without causing instability for the closed-loop control system, are well suppressed in the proposed approach as clear in the results.

The grid’s inductance variation is common in the utility grid. It occurs at the result of different events in the power grid such as the short-circuit, line outage, and etc. In the proposed approach, we considered the grid’s inductance variation as an uncertainty in the derived nominal model parameters. As a matter of fact, uncertainties may cause instability for the closed-loop control system. The same as unmodelled dynamics, in order to prevent their adverse effects on the closed-loop control system stability, it is sufficient that the loop transfer matrix be much smaller than the identity matrix in the high frequency range^27^. This condition is well satisfied in the proposed approach as clear in "Proposed dead-time compensation method" section.

### Comparison with the other works

An H_∞_ control of the grid-tied inverter is presented in^9^, where the total inductance of *L*_*t*_ = 5 mH between the inverter and the grid results in the THD = 4% for the currents injected to the grid. In the proposed approach of this paper, by employing a total inductance of *L*_*t*_ = 0.5 mH between the inverter and the grid, the THD = 5.4% for the currents injected to grid. This means that to meet the IEEE 1547 standard, i.e. THD ≤ 5%, the proposed LQG controller needs much smaller inductors in the LCL filter which is located between the inverter and the grid. This achievement is at the result of enough band-width provided by the proposed approach for the closed-loop control system.

A multivariable PI control is presented for three-phase grid-tied inverters in^28^ whose results are shown in the "Proposed dead-time compensation method" section and the "Results" section for the purpose of comparison. As shown, it resulted in the THD = 13.5% for the currents injected to grid. With the same LCL filter’s parameters, the proposed approach of this paper results in a much lower THD, i.e. THD = 5.4%, for the currents injected to grid. This is, again, at the result of enough band-width provided by the proposed LQG controller for the closed-loop control system. We want to confirm that both of the approaches in^9^ and in^28^ do not provide enough band-width for the closed-loop control system. In fact, in order to keep the closed-loop control system stable, the approaches in^9^ and in^28^ sacrifice the performance.

A dead-time compensation method is presented in^29^ for current-controlled three-phase inverters. It employs an L filter with *L* = 5mH which resulted in the currents’ THD = 6% at the 10 Hz frequency for the AC side. Considering the 50 Hz frequency in this study, the required inductors and also the achieved THD are close to those of this paper. However, the approach in^29^ does not exhibit the robust stability, since, as stated in^29^, any attempt in increasing the virtual inductor results in the closed-loop control system instability. This means that the approach in^29^ does not have appropriate stability margins while the robustness of proposed approach of this paper is proven according to the results in "Proposed dead-time compensation method" section and "Results" section.

As presented in^26^, an equivalent input disturbance-based control is employed for three-phase grid-tied inverters considering the dead-time effects. For an inductor *L* = 50 mH as the filter between the inverter and the grid, a THD = 1% is achieved in^26^ for the injected currents to grid. The need for such a large inductor shows that the approach in^26^ does not provide enough band-width for the closed-loop control system. In the proposed approach of this paper, a total inductance of *L*_*t*_ = 0.5 mH required between the inverter and the grid shows a much higher band-width provided compared to the band-width in^26^. So, the same as the approaches in^9^ and in^28^, the approach in^26^, in order to keep the closed-loop control system stable, sacrifices the performance.

## Concluding remarks and future works

In this paper, a dead-time compensation method is proposed for three-phase grid-tied inverters using an LQG-based multivariable control. Although implemented for a two-level inverter, the approach is generic; meaning that it can be employed for multilevel inverters too. The THD of injected currents to grid is dominantly determined by low-order harmonics, since high-order harmonics are well attenuated by the LCL filter. The low-order harmonics originated from either the inverter, grid, or filter are well suppressed in the proposed approach as long as their corresponding disturbances fall within the low frequency range as defined in this study. In addition to the well suppression of disturbances, the proposed approach exhibits robust stability. It means that the proposed closed-loop control system is stable in spite of uncertainties in the nominal model’s parameters and unmodelled dynamics such as the LCL filter’s capacitor branch and the PLL.

The derived nominal model for the three-phase grid-tied inverter shows that the three-phase grid-tied inverter in the synchronous reference frame is a second-order system. However, the proposed LQG control combined with the two augmented integrators form a sixth-order system. Therefore, this is the sixth-order system that must be implemented in a Digital Signal Processor (DSP) in practice. The DSP is, however, burdened by such a large computation. Therefore, it is required that the sixth-order system be appropriately reduced in terms of its order before it can be practically implemented in a DSP. Therefore, we consider an appropriate order reduction of the obtained sixth-order system as the future works. In fact, in the future works, by employing the order reduction techniques, we obtain an appropriate reduced order for the sixth-order system. The obtained controller is then implemented in a DSP and tested using the experimental set-up manufactured for the three-phase grid-tied inverter.

### Supplementary Information

Supplementary Information.

## Data Availability

The data that support the findings of this study are available from the corresponding author upon reasonable request.

## References

[1] Qiu Q, Yang F, Zhu Y, Han Q (2022). Long-horizon finite-set model predictive control for grid-connected photovoltaic inverters. Optim. Control Appl. Methods.

[2] Al-Wesabi I, Zhijian F, Hussein Farh HM (2022). Maximum power extraction and DC-Bus voltage regulation in grid-connected PV/BES system using modified incremental inductance with a novel inverter control. Sci. Rep..

[3] Jain S, Shadmand MB, Balog RS (2018). Decoupled active and reactive power predictive control for PV applications using a grid-tied quasi-Z-source inverter. IEEE J. Emerg. Sel. Top. Power Electron..

[4] Miao Z, Wei J, Guo T, Zheng M (2019). Dead-time compensation method based on field oriented control strategy. IOP Conf. Ser..

[5] Moghadasi, A. *et al.* A simplified power control approach with reliable axis decoupling capability for three-phase current source inverter. In *2016 IEEE International Conference on Power Electronics, Drives and Energy Systems (PEDES)* 1–7 (IEEE, 2016).

[6] Lopez-Santos O, Garcia G, Martinez-Salamero L, Avila-Martinez JC, Seguier L (2017). Non-linear control of the output stage of a solar microinverter. Int. J. Control.

[7] Altin N, Ozdemir S, Komurcugil H, Sefa I (2018). Sliding-mode control in natural frame with reduced number of sensors for three-phase grid-tied LCL-interfaced inverters. IEEE Trans. Ind. Electron..

[8] Ji Y, Yang Y, Zhou J, Ding H, Guo X, Padmanaban S (2019). Control strategies of mitigating dead-time effect on power converters: An overview. Electronics.

[9] Wang Y, Wang J, Zeng W, Liu H, Chai Y (2018). H∞ robust control of an LCL-type grid-connected inverter with large-scale grid impedance perturbation. Energies.

[10] Chowdhury MA (2016). Dual-loop H∞ controller design for a grid-connected single-phase photovoltaic system. Sol. Energy.

[11] Bhadu M, Senroy N, Narayan Kar I, Sudha GN (2016). Robust linear quadratic gaussian-based discrete mode wide area power system damping controller. IET Gener. Trans. Distrib..

[12] Baumgartner T, Kolar JW (2014). Multivariable state feedback control of a 500000-r/min self-bearing permanent-magnet motor. IEEE/ASME Trans. Mechatron..

[13] Wang J, He R (2018). Varying charge voltage in the steps control method of ABS for in-wheel motors driven electric vehicles based on an improved LQG scheme. IEEE Access.

[14] Zhu Q, Ding J, Yang M (2018). LQG control based lateral active secondary and primary suspensions of high-speed train for ride quality and hunting stability. IET Control Theory Appl..

[15] Andani, M. T., Pourgharibshahi, H., Ramezani, Z. & Zargarzadeh, H. Controller design for voltage-source converter using LQG/LTR. In *2018 IEEE Texas Power and Energy Conference (TPEC)* 1–6 (IEEE, 2018).

[16] Angelico BA, Toriumi FY, Barbosa FS, Neves GPD (2017). On guaranteeing convergence of discrete LQG/LTR when augmenting it with forward PI controllers. IEEE Access.

[17] Florescu A, Bratcu AI, Munteanu I, Rumeau A, Bacha S (2014). LQG optimal control applied to on-board energy management system of all-electric vehicles. IEEE Trans. Control Syst. Technol..

[18] Al-Digs A, Dhople SV, Chen YC (2018). Measurement-based sparsity-promoting optimal control of line flows. IEEE Trans. Power Syst..

[19] Pérez-Ibacache R, Silva CA, Yazdani A (2018). Linear state-feedback primary control for enhanced dynamic response of AC microgrids. IEEE Trans. Smart Grid.

[20] Ouammi A, Dagdougui H, Sacile R (2014). Optimal control of power flows and energy local storages in a network of microgrids modeled as a system of systems. IEEE Trans. Control Syst. Technol..

[21] Panigrahi R, Subudhi B, Panda PC (2015). A robust LQG servo control strategy of shunt-active power filter for power quality enhancement. IEEE Trans. Power Electron..

[22] Huerta F (2011). LQG servo controller for the current control of LCL grid-connected voltage-source converters. IEEE Trans. Ind. Electron..

[23] Huerta F, Perez J, Cóbreces S, Rizo M (2018). Frequency-adaptive multiresonant LQG state-feedback current controller for LCL-filtered VSCs under distorted grid voltages. IEEE Trans. Ind. Electron..

[24] Benrabah A, Xu D, Gao Z (2018). Active disturbance rejection control of LCL-filtered grid-connected inverter using pade approximation. IEEE Trans. Ind. Appl..

[25] Saleem M, Choi KY, Kim RY (2019). Resonance damping for an LCL filter type grid-connected inverter with active disturbance rejection control under grid impedance uncertainty. Int. J. Electr. Power Energy Syst..

[26] Fang L, Zhen F, Qianyi L, Runmin Z (2021). Equivalent input disturbance-based control design for three phase dual-stage grid-tied photovoltaic system considering dead time effect. Front. Energy Res..

[27] Maciejowski, J. M. Electronic systems engineering series. In *Multivariable feedback design* (Addison-Wesley, 1989).

[28] Mazaheri, A., Barati, F. & Ghavipanjeh, F. Multi-variable PI control design for grid-tied three-phase PV inverters. In *2019 Iranian Conference on Renewable Energy & Distributed Generation (ICREDG)* 1–5 (IEEE, 2019).

[29] Putri AI, Rizqiawan A, Rachmildha TD, Haroen Y, Dahono PA (2020). Minimization of dead-time effect in current-controlled three-phase PWM inverters by using virtual inductor. Int. J. Electr. Eng. Inf..

