# Supplementary material for: Dead-time compensation in three-phase grid-tied inverters using LQG multivariable control
Source: Sci Rep. 2023 Sep 8;13:14851. doi: 10.1038/s41598-023-41944-2 (PMC10491623; doi:10.1038/s41598-023-41944-2)
Supplement: Supplementary file 1 — Supplementary Information. [file 41598_2023_41944_MOESM1_ESM.docx]

**Appendix**

We developed a detailed model in MATLAB/Simulink for the manufactured three-phase grid-tied inverter. It is shown in Fig. (A-1). The implemented inverter is a two-level three-phase one with IGBT power switches. The parameters of IGBT power switches in the detailed model are set according to the datasheet of the BSM50GP120 IGBT power module employed in the experimental set-up. It is commanded by the SPWM in which the dead-time is included to protect the IGBTs’ legs from short-circuit. The implemented data for the switching frequency and the dead-time are as in Table 1. In order to suppress high-order harmonics produced by the inverter, an LCL filter is implemented between the inverter and the grid. Having a compact filter while satisfying the THD requirements is an important goal followed in this study. The implemented LCL filter has a Y connection whose neutral point is connected to the middle point of the DC link. Note that, the DC link is divided into two equal voltages by employing two equal capacitors. The LCL filter is equipped with a damping resistor whose role is to provide an appropriate damping at the filter’s resonance frequency.

A PLL is implemented for calculating the angle of grid’s voltage vector. In fact, it calculates the angle of positive-sequence component in an un-balanced grid. It, of course, has dynamics in following the grid’s angle whose effects are seen in the results section. The calculated angle is then employed in the *abc/dq* or *dq/abc* transformations as shown in Fig. (A-1). We want to confirm that the role of PLL is essential in the closed-loop control system of the grid-tied inverter. Note that, the *abc/dq* transformation is the Park transformation in which the time-domain components of a three-phase system in an *abc* reference frame are converted to the *direct* and *quadrature* components in a rotating reference frame. The rotating reference frame implemented in this study is a synchronous reference frame aligned with the grid’s voltage vector.

We implemented a current-controlled three-phase inverter in this study in which the inverter’s output currents are appropriately controlled in the synchronous reference frame. The reference inputs are I^*^_d_ and I^*^_q_ which are calculated according the reference active and reactive powers as shown before. The reference active and reactive powers are received from the supervisory control in a grid-tied DC microgrid. As seen, the inverter is connected to the utility grid which is at the distribution level. The utility grid is connected to the transmission lines through a power transformer. The inductances shown between the LCL filter and the utility grid represent the grid’s inductances at the PCC.


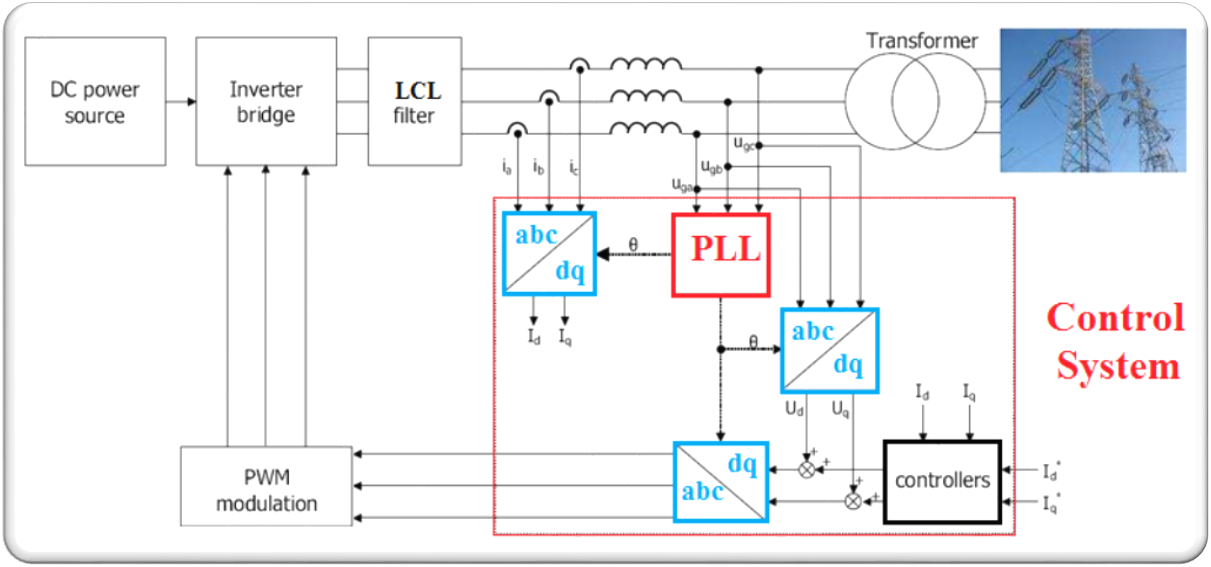


**Figure (A-1)**. Detailed model developed in MATLAB/Simulink for the manufactured three-phase grid-tied inverter

Table 2. Classifications of different phenomena in three-phase grid-tied inverter

In Fig. (A-2), the manufactured experimental set-up is shown. High band-width, high accuracy, and isolated measurements are provided for the grid’s three-phase currents and voltages in the experimental set-up. A 320F28335 Texas Instruments DSP board is considered in the experimental set-up for implementing the inverter’s control system. The SPWM is implemented in the DSP board too whose outputs are connected to the IGBTs’ driver ICs mounted on the main board. In order to protect both of the DC and AC sides against over voltages, metal-oxide varistors are employed at both sides. The DC link is fed from a rooftop PV array whose maximum achievable power varies according to the weather conditions. Therefore, in the experimental set-up, the reference active power varies according to the weather conditions. Also, the reference reactive power can be set to zero in order to dedicate the inverter’s nominal apparent power to the active power. Note that, the zero reactive power means that the *PF* =1 at the PCC.

Three-Phase AC Connection

DC Link Capacitors

DSP Board

LCL Filter

IGBT Power Module, Heat Sink & Fan

DC Connection


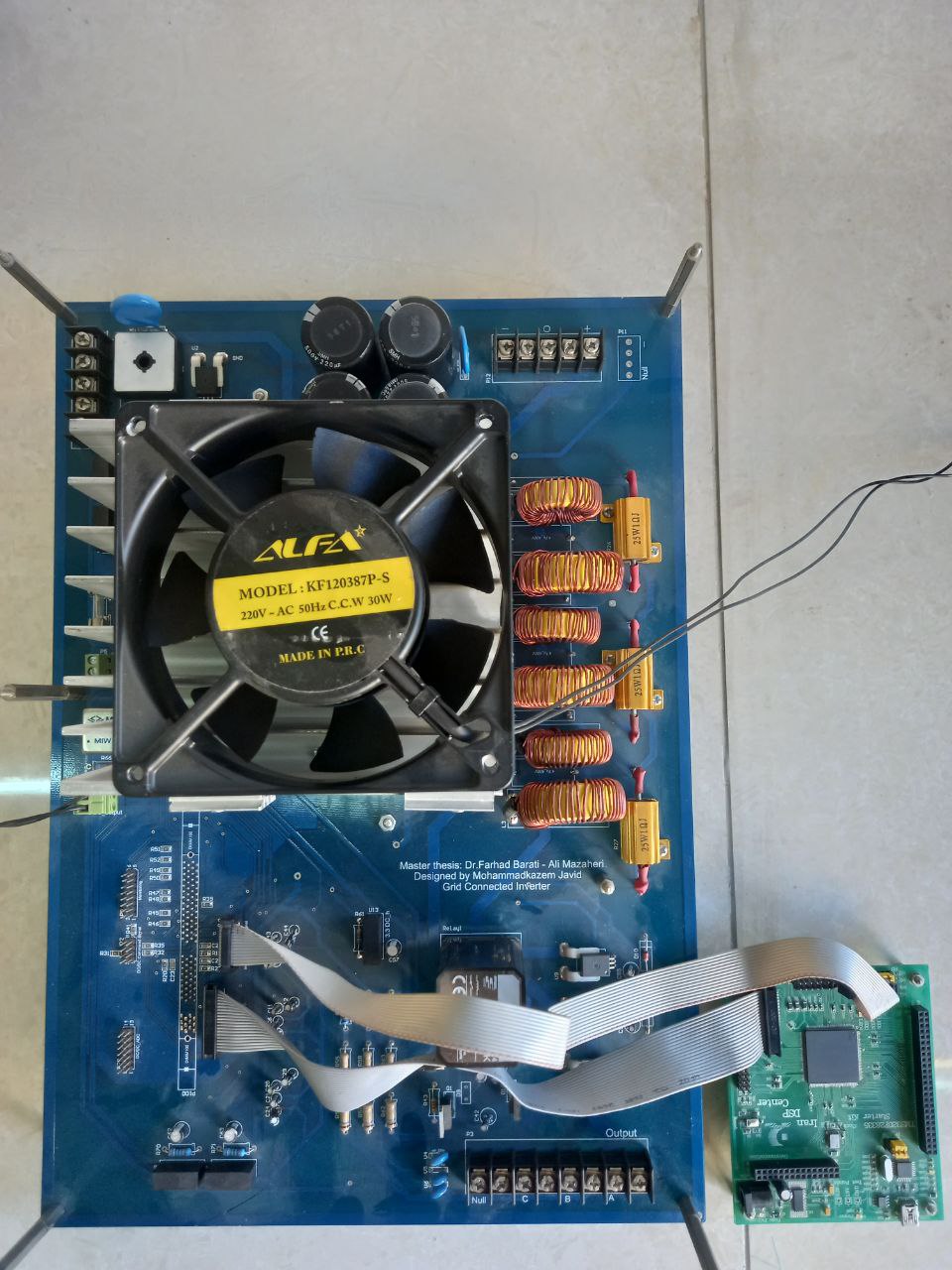


**Figure (A-2)**. Manufactured experimental set-up for three-phase grid-tied inverter

Table 2. Classifications of different phenomena in three-phase grid-tied inverter
